# Performance analysis of remote photoplethysmography deep filtering using long short-term memory neural network

**DOI:** 10.1186/s12938-022-01037-z

**Published:** 2022-09-19

**Authors:** Deivid Botina-Monsalve, Yannick Benezeth, Johel Miteran

**Affiliations:** Univ. Bourgogne Franche-Comté, ImViA EA7535 Dijon, France

**Keywords:** Remote photoplethysmography, rPPG filtering, Signal enhancement, Long short-term memory, MMSE-HR, VIPL-HR, COHFACE, LSTM-DF

## Abstract

**Background:**

Remote photoplethysmography (rPPG) is a technique developed to estimate heart rate using standard video cameras and ambient light. Due to the multiple sources of noise that deteriorate the quality of the signal, conventional filters such as the bandpass and wavelet-based filters are commonly used. However, after using conventional filters, some alterations remain, but interestingly an experienced eye can easily identify them.

**Results:**

We studied a long short-term memory (LSTM) network in the rPPG filtering task to identify these alterations using many-to-one and many-to-many approaches. We used three public databases in intra-dataset and cross-dataset scenarios, along with different protocols to analyze the performance of the method. We demonstrate how the network can be easily trained with a set of 90 signals totaling around 45 min. On the other hand, we show the stability of the LSTM performance with six state-of-the-art rPPG methods.

**Conclusions:**

This study demonstrates the superiority of the LSTM-based filter experimentally compared with conventional filters in an intra-dataset scenario. For example, we obtain on the VIPL database an MAE of 3.9 bpm, whereas conventional filtering improves performance on the same dataset from 10.3 bpm to 7.7 bpm. The cross-dataset approach presents a dependence in the network related to the average signal-to-noise ratio on the rPPG signals, where the closest signal-to-noise ratio values in the training and testing set the better. Moreover, it was demonstrated that a relatively small amount of data are sufficient to successfully train the network and outperform the results obtained by classical filters. More precisely, we have shown that about 45 min of rPPG signal could be sufficient to train an effective LSTM deep-filter.

**Supplementary Information:**

The online version contains supplementary material available at 10.1186/s12938-022-01037-z.

## Background

Electrocardiography (ECG) and photoplethysmography (PPG) are two methods that are used to measure different physiological parameters of the body, such as heart rate (HR) and heart rate variability (HRV); with which it is possible to monitor the behavior of the heart. ECG is a method that measures the electrical field caused by heart activity. On the other hand, PPG measures variations in light absorption in tissues due to the pulsatile nature of the cardiovascular system and the variation in blood volume [1]. Heart rate monitoring can be conducted by invasive methods such as pulmonary artery catheterization [2], and noninvasive methods classified as contact-based and non-contact-based. ECG and PPG methods perform contact-based HR measurements, and they may cause hygiene issues, discomfort, or even be impossible on fragile skins. Due to these possible disadvantages, in [3], Verkruysse et al., demonstrated that PPG signals could be measured remotely from a standard video camera, using ambient light as an illumination source. This technique, known as remote photoplethysmography offers the advantage of measuring the same parameters as PPG in an entirely remote way. In fact, rPPG is the non-contact equivalent to the reflective mode of PPG using ambient light as a source and a camera as a receptor. The light reflected by the skin is then estimated by capturing subtle skin color variations by the camera as blood volume changes. Several images and signal processing steps allow to obtain a pulse signal, also called the rPPG signal.

With rPPG or PPG signals, several biomedical parameters can potentially be measured: heart rate, pulse rate variability, breathing rate, vascular occlusion, peripheral vasomotor activity, and blood pressure by pulse transit time [4, 5]. Likewise, the applications are multiple, and some examples are mixed reality [6], physiological measurements of car drivers [7], living skin segmentation [8], control of vital signs in the elderly and newborns [9], and face anti-spoofing [10] to name a few.

The first approach implemented to estimate rPPG signals was only based on the green channel [3]. Then, approaches based on blind source separation techniques were proposed, e.g., PCA, ICA, EVM, PVM [11–15], and others based on a light tissue interaction model to determine a projection vector, e.g., PbV, POS, and Chrom [16–18]. In-depth state-of-the-art reviews of these rPPG signal estimation techniques are presented in [19–21]. More recently, some methods have adopted the strong modeling ability of deep neural networks for physiological measurements from video sequences [22–24]. The main advantages of these methods are that it allows achieving good results without the need for the designer to analyze the problem in-depth [25]. With the hand-crafted methods, it is necessary to detect and track the region of interest (ROI) through the frames, combine the RGB channels, filter them and estimate the physiological parameters such as heart rate or respiration rate. In the deep-learning-based measurement, on the other hand, a pipeline-based framework is no longer necessary. Consequently, deep-learning-based methods are less prone to error propagation in their pipeline. However, recent work has focused on heart rate measurement performance rather than understanding [25]. Subsequently, the limitations of the system are not always clear. Besides, it is well known that the training dataset used is critical.

**Fig. 1:**
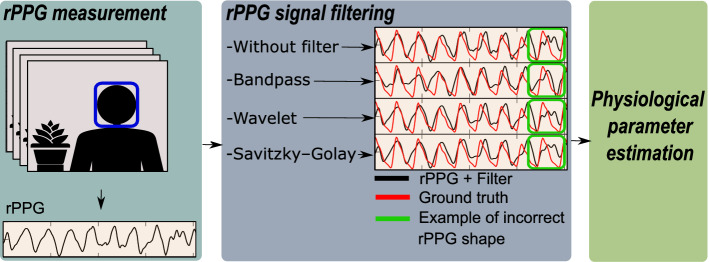
Conventional pipeline for video-based physiological parameters estimation. The rPPG signal is acquired from a video then a classical filtering method is used. The physiological parameters are calculated from the filtered signal. After the filtering, some irregular shapes of the signal remain (ground truth is the blood volume pulse (BVP) signal measured with the CONTEC CMS60C pulse oximeter)

Usually, the rPPG signal estimated from any of these methods is noisy due to the estimation technique, illumination variations, internal noise of the digital camera, and motion. Consequently, once the rPPG signals are acquired, unnecessary information such as frequencies out of the normal physiological range of interest are removed using a filtering process. The smoothing operation is commonly performed by a bandpass filter (BP) [12, 26–30], sometimes by a wavelet-based filter (WV) [13, 31–34], and recently, by the Savitzky–Golay filter (SG) [35–37]. Although these methods do smooth the rPPG signals, they do not necessarily remove particular signal alterations, which can, however, be easily identified by experts. Figure 1 shows the conventional pipeline used in the literature. The rPPG signal extracted from the video is smoothed through a classical filter for subsequent estimation of physiological parameters. However, even after the filtering process, irregular shapes of the rPPG signal are observed (see signal parts in green boxes in Fig. 1). The remaining alterations in the rPPG signal may affect the accuracy of heart rate measurements, but more gravely, avoid further advanced analysis of rPPG signals that can be based on peaks detection and pulse shape characteristics on temporal signals. For example, authors in [38] measured HRV by estimating the time elapsed between consecutive peaks of an rPPG signal to estimate emotional states. In this particular application, a noisy rPPG signal with false peaks would lead to erroneous measurement of HRV and, consequently, a misinterpretation of the emotional state. Therefore, there is a need to improve the accuracy of heart rate measurement and the rPPG signal quality. In this work, we want to benefit from the advantages of deep-learning-based methods, specifically by carrying out an in-depth study on the use of recurrent neural networks to improve the filtering of rPPG signals, proposing different protocols that allow a better understanding of these networks in the filtering application.

Recurrent Neural Networks (RNNs) are commonly used in applications where the structure embedded in the data sequences transfers valuable information, much like an expert would identify the wrong peaks in an rPPG signal. However, these networks show the well-known *vanishing/exploding* gradients problem during backpropagation, and because of this, RNNs cannot store information for a very long time. The long short-term memory (LSTM), on the other hand, is a network with memory blocks in its recurrent connections, saving information for more extended periods, avoiding the problem present in RNNs networks [23, 39]. The LSTM networks have been used successfully in the literature to process medical signals such as ECG, proving the potential of this type of network [40–42]. In [43], the authors propose a method of PPG denoising based on a bidirectional recurrent denoising auto-encoder (BRDAE) to retain the recurrent information in the PPG signal. The network training and testing are performed on an artificial noise-augmented PPG database, along with additional PPG signals acquired from subjects during their daily routine. Slapnicar et al. [44] propose an LSTM-based method to enhance reconstructed rPPG signals obtained by the POS method. For this purpose, they use a bandpass filter and a two-step wavelet filter to finally use a 2-layer LSTM network, using a many-to-one sequence-to-sequence approach. Although the results are satisfactory, using two classical filtering methods before using LSTM networks is necessary, when a single LSTM filtering stage could be sufficient. To the best of our knowledge, the only approach used in the literature to filter rPPG signals directly with an LSTM-based filter is the one proposed in [45], where the authors proposed a two-layer LSTM model to filter the rPPG signals in the MMSE-HR database. The authors affirm that a deep neural network requires thousands of training data, and their way of facing this problem is to train the network in synthetic signals based on sine signals with random Gaussian noise and then fine-tune the network in the database. However, although the proposed synthetic signals manage to simulate the rPPG signals with frequencies corresponding to the heart rate, they do not contain the characteristic shape of the signals in a real acquisition scenario, preventing the network from learning the subtle details of the signal such as saving the dicrotic notch shape or suppressing double peaks as described in [46]. Table 1 shows the publications mentioned above organized according to the type of signal and the method used for their respective objective.

**Table 1:** Publications related to classical and machine learning methods for filtering ECG, PPG, and rPPG signals

Publications	Signal	Method	Objective
[12, 26–30]	rPPG	Bandpass	Filtering
[13, 31–34]	rPPG	Wavelet	Filtering
[35–37]	rPPG	Savitzky–Golay	Filtering
[40–42]	ECG	LSTM	Classification
[43]	PPG	BRDAE	Filtering
[44]	rPPG	Bandpass+wavelet+LSTM	Enhance reconstructed rPPG
[45]	rPPG	LSTM	Filtering

In summary, these previous works filter PPG or rPPG signals with different approaches, where the authors agree that it is necessary to obtain these signals with the best possible quality. Although these works already generate considerable progress in extracting quality rPPG signals, there is still room for improvement. In addition, despite the excellent performances of biomedical signal filtering techniques based on machine learning (e.g., [43, 47]), they are still rarely used compared to more classical techniques (like bandpass, etc.). We believe that this is due to the lack of studies investigating the advantages, limitations, and sensitivity of these methods. There is no in-depth study on the specific aspect of neural-network-based filtering, namely the influence of amount of training data, influence of input signal quality and influence of dataset used for training and testing. To our knowledge, our paper is the first to present such a study on biomedical signals and, in particular, on rPPG signals.

Indeed, in deep-learning-based applications, the amount of data used during the training of a network plays a fundamental role in its performance. For example, in computer vision applications, generalization tends to improve with the size of the training sets [48]. However, there is no definitive answer to whether the amount of data during training improves all deep-learning applications. For this reason, it is critical to define in each deep-learning-based application if there is a dependency on the amount of data used during the training. In this way, it is possible to better understand the limitations and sensitivities of these networks.

In [47], we proposed the LSTM-DF filter to be used on rPPG signals estimated by the PVM method in the MMSE database [49], where we considered the filtering of these signals as a sequence-to-sequence regression problem, so we entered multiple inputs (samples) to the LSTM-DF filter to generate multiple outputs. This sequence-to-sequence approach is known as many-to-many (LSTM-MTM). The other sequence-to-sequence approach is the many-to-one (LSTM-MTO), where multiple inputs give a single output, like the one used in [44]. In this article, we analyze the performance of LSTM recurrent networks in rPPG signal filtering. Using the LSTM deep-filter, we develop an in-depth study of the network performance, adding experiments and approaches to determine the limitations and sensitivities of using these networks, allowing us to understand the best configuration to train an LSTM-based model to filter rPPG signals.

To the best of our knowledge, this is the first work where the performance of LSTM recurrent networks for rPPG signal filtering is studied. The main contributions of this work include (1) using three public-domain databases in intra-dataset and cross-dataset scenarios, we experimentally demonstrate the advantages of using LSTM networks in rPPG signal filtering. (2) We present a comparison between two sequence-to-sequence LSTM-based filters, namely MTO and MTM approaches, where MTM proved to be the most successful method. (3) We compare the use of the LSTM-based filter with six state-of-the-art rPPG signal estimation methods: PVM, POS, PbV, G-R, Chrom, and Green, where we observed high stability of the LSTM-based filter concerning different input data obtained with different methods. (4) Interestingly, we also show that a relatively small dataset can be enough to train the network and that there is a significant dependence on the signal-to-noise-ratio average on the training signals.

The remainder of the paper is organized as follows: in “Results” and “Discussion” sections, we present the outcome of the proposed experiments and a discussion. After “Conclusions” section, in “Methods” section, we explain the three public databases used, the protocols proposed to understand the limitations and sensitivities of the LSTM-based network, and the two approaches of the LSTM-based filter, namely MTO and MTM. Finally, we show the evaluation metrics and the implementation details of the network used.

## Results

In this section, we present the results of a series of experiments where we compare three classical filters: bandpass, wavelet, and Savitzky–Golay, with two LSTM-based deep-filter approaches—MTO and MTM. We use fivefold subject-independent cross-validation on the MMSE-HR, VIPL-HR, and COHFACE databases. To evaluate the filtering methods rigorously, we use the mean absolute error (MAE) and the Pearson correlation coefficient (*r*) to describe the quality of the heart rate estimations, and also the signal-to-noise ratio (SNR), and the template match correlation (TMC), to describe the signal quality. Metrics are calculated with a 15-s sliding window with a 0.5-s step. SNR, TMC, and *r* are to be maximized, while MAE has to be minimized For ease of data visualization, the MAE metric is presented in the figures with the vertical axis inverted; this is done so that the best performances for the four metrics are always at the top of the graphs. Best results in experiments *intra-dataset* and *cross-dataset* are presented in bold red. Metrics, databases, and protocols are explained in detail in “Methods” section.

### Intra-dataset

**Fig. 2:**
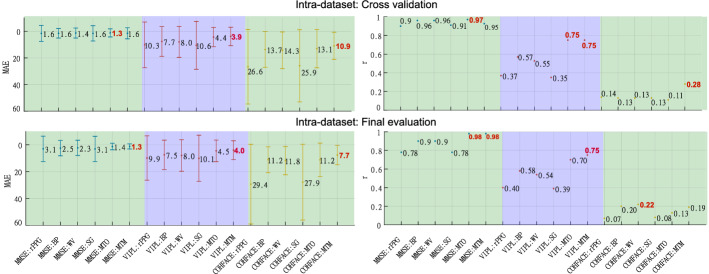
Intra-dataset heart rate measurement evaluation. Results of the filters: bandpass (BP), wavelet (WV), Savitzky–Golay (SG), LSTM-DF MTO and MTM. Best values are presented in bold red

**Fig. 3:**
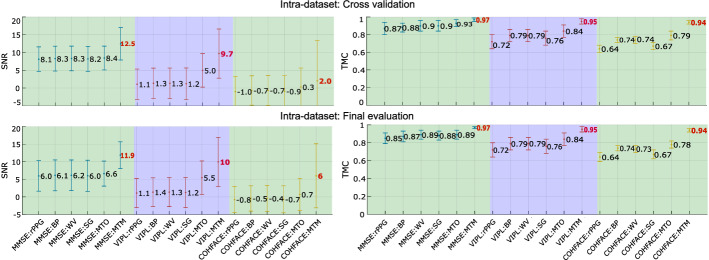
Intra-dataset signal quality evaluation. Results of the filters: bandpass (BP), wavelet (WV), Savitzky–Golay (SG), LSTM-DF MTO and MTM. Best values are presented in bold red

Figure 2 presents the results associated with the heart rate measurement metrics in the MMSE-HR, VIPL-HR, and COHFACE databases. On the left are the MAE values, and on the right *r*, on the upper part are the results of the k-fold cross-validation, and on the lower part is the final evaluation. Figure 3 depicts the metrics related to the signal quality, in the left part the SNR coefficient and the right part the TMC coefficient.

**Fig. 4:**
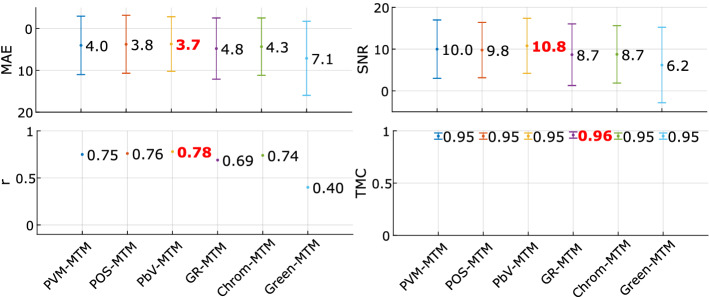
rPPG methods comparison with LSTM-DF MTM. Comparison between rPPG estimation methods after using LSTM-based filter many-to-many approach in a intra-dataset scenario

Table 2 contains the rPPG-SNR average of the signals acquired in each method, where the same 2,256 subjects have a total rPPG signal duration of 1,114.5 min for the six sets. As the second part of this experiment, six state-of-the-art methods for rPPG signal estimation: PVM, POS, PbV, G-R, Chrom, and Green, were chosen to be applied to the VIPL-HR images. Figure 4 compares the six state-of-the-art rPPG signal acquisition methods after using the MTM filter. Tables 3 and 4 present the cross-validation and final evaluation results, respectively, for the six methods (best results in bold).

**Table 2:** RPPG-SNR average in the VIPL-HR signals acquired by the methods: PVM, POS, PbV, G-R, Chrom and Green

	PVM-VIPL	POS-VIPL	PbV-VIPL	GR-VIPL	Chrom-VIPL	Green-VIPL
rPPG-SNR [dB]	$1.07\pm 4.25$	$0.32\pm 3.71$	$-0.29\pm 3.3$	$-0.51\pm 4$	$-0.92\pm 3.4$	$-1.36\pm 4.05$

**Table 3:** Intra-dataset, cross-validation results for rPPG signals estimated from VIPL-HR dataset by PVM, POS, PbV, G-R (GR), Chrom and Green methods

	PVM-VIPL						POS-VIPL						PbV-VIPL					
	rPPG	BP	WV	SG	MTO	MTM	rPPG	BP	WV	SG	MTO	MTM	rPPG	BP	WV	SG	MTO	MTM
MAE	10.25	7.71	8.03	10.57	4.4	**3**.**93**	7.45	6.42	6.26	7.23	3.98	**3**.**71**	6.92	6.31	6.12	6.24	4.06	**3**.**94**
r	0.37	0.57	0.55	0.35	**0**.**75**	**0**.**75**	0.57	0.64	0.65	0.58	**0**.**79**	0.75	0.57	0.62	0.63	0.64	**0**.**79**	0.73
SNR	1.07	1.34	1.33	1.21	4.97	**9**.**69**	0.58	0.74	0.81	1.05	4.72	**9**.**76**	0.16	0.32	0.38	0.67	4.92	**9**.**58**
TMC	0.72	0.79	0.79	0.76	0.84	**0**.**95**	0.55	0.71	0.7	0.7	0.8	**0**.**95**	0.57	0.75	0.74	0.73	0.79	**0**.**95**

	GR-VIPL						Chrom-VIPL						Green-VIPL					
	rPPG	BP	WV	SG	MTO	MTM	rPPG	BP	WV	SG	MTO	MTM	rPPG	BP	WV	SG	MTO	MTM
MAE	18.31	12.78	12.71	17.68	5.23	**4**.**87**	9.23	8.39	8.06	8.09	**4**.**52**	4.72	26.43	18.02	18.16	26.01	9.3	**7**.**14**
r	0.18	0.34	0.34	0.18	**0**.**68**	0.67	0.47	0.5	0.53	0.54	**0**.**75**	0.67	0.08	0.06	0.06	0.07	0.24	**0**.**41**
SNR	− 1.13	− 0.9	− 0.8	− 0.9	3.53	**8**.**35**	− 0.48	− 0.32	− 0.26	0.04	3.83	**8**.**43**	− 2.71	− 2.46	− 2.38	− 2.61	1.36	**5**.**66**
TMC	0.59	0.74	0.74	0.71	0.79	**0**.**95**	0.53	0.7	0.69	0.68	0.77	**0**.**95**	0.55	0.71	0.7	0.66	0.75	**0**.**94**

**Table 4:** Intra-dataset, final evaluation results for rPPG signals estimated from VIPL-HR dataset by PVM, POS, PbV, G-R (GR), Chrom and Green methods

	PVM-VIPL						POS-VIPL						PbV-VIPL					
	rPPG	BP	WV	SG	MTO	MTM	rPPG	BP	WV	SG	MTO	MTM	rPPG	BP	WV	SG	MTO	MTM
MAE	9.91	7.49	8.02	10.12	4.51	**4**.**02**	7.16	6.26	6.14	7.03	3.85	**3**.**76**	6.79	6.28	6.04	6.21	4.05	**3**.**7**
r	0.4	0.58	0.54	0.39	0.7	**0**.**75**	0.56	0.62	0.63	0.58	**0**.**81**	0.76	0.6	0.64	0.64	0.65	**0**.**78**	**0**.**78**
SNR	1.08	1.35	1.34	1.24	5.48	**9**.**98**	0.6	0.76	0.83	1.08	5.19	**9**.**76**	0.19	0.35	0.41	0.71	5.04	**10**.**78**
TMC	0.72	0.79	0.79	0.76	0.84	**0**.**95**	0.55	0.72	0.71	0.71	0.81	**0**.**95**	0.57	0.75	0.74	0.73	0.78	**0**.**95**

	GR-VIPL						Chrom-VIPL						Green-VIPL					
	rPPG	BP	WV	SG	MTO	MTM	rPPG	BP	WV	SG	MTO	MTM	rPPG	BP	WV	SG	MTO	MTM
MAE	18.75	12.93	12.98	18.34	**4**.**79**	**4**.**79**	9.27	8.5	8.27	8.12	4.37	**4**.**33**	25.73	17.72	17.79	25.43	10	**7**.**12**
r	0.15	0.36	0.33	0.15	**0**.**72**	0.69	0.52	0.56	0.56	0.57	**0**.**77**	0.74	0.06	0.07	0.05	0.05	0.23	**0**.**4**
SNR	− 1.22	− 1	− 0.92	− 1	3.87	**8**.**66**	− 0.55	− 0.4	− 0.33	− 0.04	4.27	**8**.**74**	− 2.72	− 2.46	− 2.39	− 2.62	1.32	**6**.**18**
TMC	0.59	0.74	0.73	0.71	0.81	**0**.**96**	0.53	0.71	0.69	0.68	0.79	**0**.**95**	0.55	0.71	0.7	0.66	0.74	**0**.**95**

### Cross-dataset

**Fig. 5:**
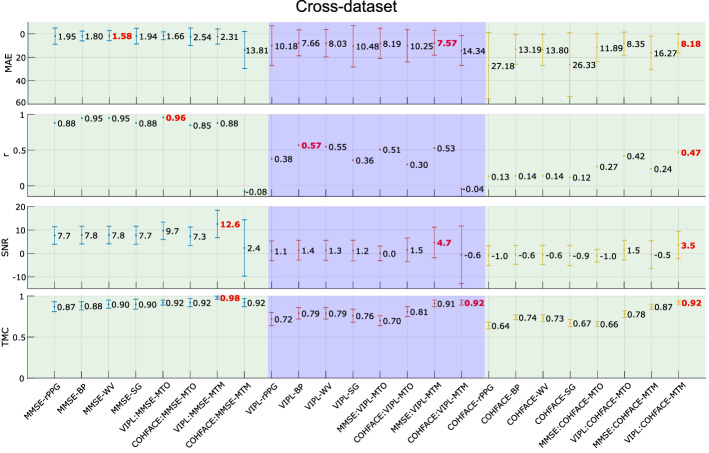
Cross-dataset. Results of MAE, *r*, SNR and TMC metrics in the *cross-dataset* experiment

Figure 5 shows the results of the metrics related to the estimation of the heart rate and signal quality, during the *cross-dataset* experiment, for the MMSE-HR, VIPL-HR, and COHFACE databases. The first row contains the MAE values, the second one is *r*, the third one with the SNR coefficient, and the fourth one with the TMC coefficient.

### Amount of training data

**Fig. 6:**
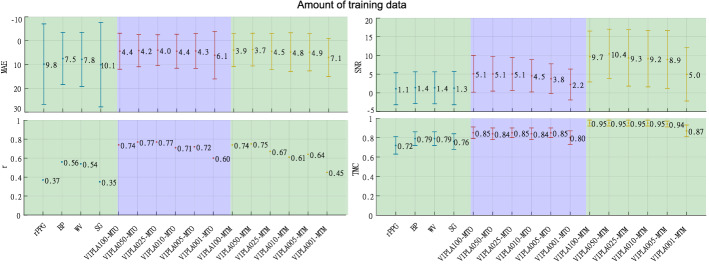
Amount of training data. Results in the *amount of training data* experiment

Table 5 contains the parameters of the rPPG signals used in this experiment, the average rPPG-SNR, the total duration of the signals in minutes, and the number of signals. Note how the average rPPG-SNR is similar for the seven sub-sets; this ensures that the results obtained in this experiment are not affected by the quality of the signals but only by the quantity. Figure 6 depicts the results of this experiment. On the left side of the figure are the metrics MAE and *r*, and on the right side, SNR along with TMC. MAE, SNR, and TMC are the metric average with its confidence interval.

**Table 5:** Parameters of the rPPG signals in *amount of training data*

	VIPLA100	VIPLA050	VIPLA025	VIPAL010	VIPLA005	VIPLA001	VIPLB
rPPG-SNR [dB]	$1.27\pm 4.07$	$1.25\pm 4.04$	$1.21\pm 4.0$	$1.15\pm 4.13$	$0.99\pm 4.11$	$1.03\pm 3.92$	$1.09\pm 4.28$
Duration [min]	891.78	446.11	223.5	88.87	45.1	9.04	222.7
Number of signals	1084	902	451	180	90	18	452

### rPPG-SNR dependence

**Fig. 7:**
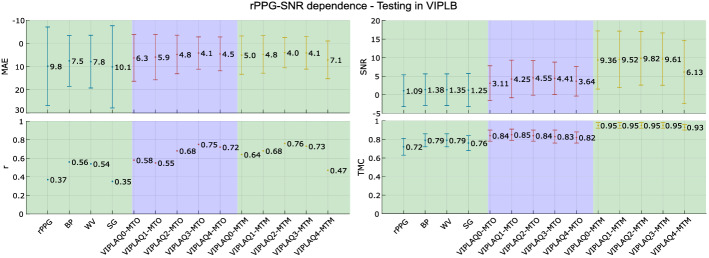
rPPG-SNR dependence 1. Results of MAE, *r*, SNR and TMC metrics in the *rPPG-SNR dependence* experiment. Training in VIPLAQ0, VIPLAQ1, VIPLAQ2, VIPLAQ3, and VIPLAQ4, and testing in VIPLB

The characteristics of the rPPG signals used in this experiment are presented in Table 6, the average SNR of the rPPG signals, the total duration, and the number of signals. Note how the duration and number of signals for the VIPLA sets are balanced, while the more considerable variation is found in rPPG-SNR due to the quality of the videos and the rPPG estimation method. Figure 7 presents the metrics MAE and *r* on the left and SNR with TMC on the right for the first part of this experiment, where we trained the LSTM network in VIPLAQi, i=[0,1,2,3,4] and tested in VIPLB.

**Table 6:** Characteristics of the rPPG signals in *rPPG-SNR dependence* protocol

	VIPLAQ0	VIPLAQ1	VIPLAQ2	VIPLAQ3	VIPLAQ4	VIPLB
rPPG-SNR [dB]	$6.42\pm 2.48$	$5.64\pm 3.15$	$1.89\pm 6.23$	$0.73\pm 5.56$	$-2.69\pm 2.66$	$1.09\pm 4.28$
Duration [min]	195.22	196.42	195.22	198.3	197.56	222.7
Number of signals	400	400	397	407	400	452

**Fig. 8:**
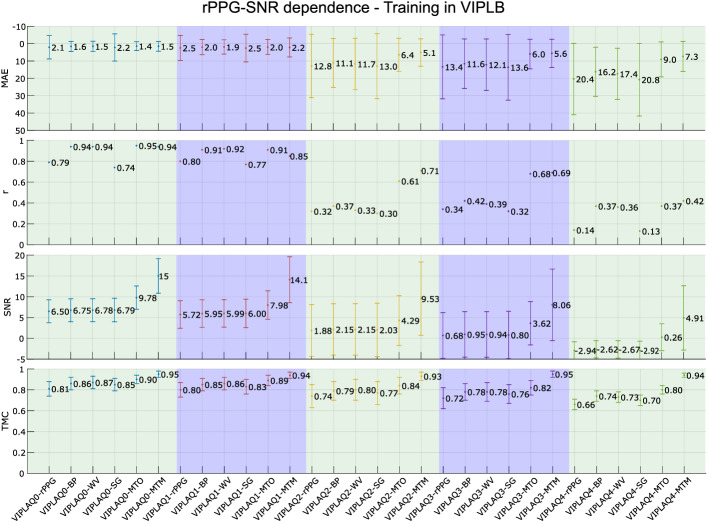
rPPG-SNR dependence 2. Results of MAE, *r*, SNR and TMC metrics in the *rPPG-SNR dependence* experiment. Training in VIPLB and testing in VIPLAQ0, VIPLAQ1, VIPLAQ2, VIPLAQ3, and VIPLAQ4 sub-sets

As the second part of this experiment, the LSTM network is trained in VIPLB to be tested in VIPLAQi, i=[0,1,2,3,4]. Figure 8 depicts the results given by this second part of the current experiment. The first row has the MAE metric, the second one *r*, the third one SNR, and the final one TMC.

In the following section, we will discuss the results presented.

## Discussion

Based on the tables and figures presented in ”Results” section, this section analyzes in detail the behavior of the LSTM network in each of the proposed experiments.

In the *intra-dataset* experiment, Figs. 2 and 3 show that MTO and MTM present good results in the MMSE-HR database compared with classical filters. The BP and WV filters have results very close to those obtained by LSTM-based filter approaches in MAE and *r*. We believe this is due to the good quality of the videos and scenarios within the database, verifiable in the minimal difference in MAE and *r* from the raw rPPG signal and all the filtering methods analyzed. In the VIPL-HR database, the MTO and MTM approaches always have the best results, being the MTM approach the best one. The good performance of the LSTM-based filter in this particular dataset is due to a large number of signals available, allowing the network to learn in a greater variety of signals than the MMSE-HR and COHFACE databases, where there is less variety of the signals. In the COHFACE dataset, the MTM approach is the LSTM-based filter that gives the best results, with the only exception of *r* in the final evaluation, where the value $r=0.19$ is very close to the best result $r=0.22$. The poor values shown in the raw signal rPPG in MAE and *r* can be associated with the compression of the videos present in this specific database. It is important to note that for the three databases, the best SNR and TMC values are those acquired by the two approaches of the LSTM network. However, MTM increases the quality of the signals to a greater extent.

In Tables 3 and 4, it can be seen that the LSTM-based filter always gives the best results compared with the filtering methods used in the literature. In MMSE, VIPL, and COHFACE databases, SNR and TMC metrics show significant improvements in signal quality when using the MTM approach. On the other hand, the MTO approach is only slightly higher than MTM in the MAE and *r* metrics. Finally, Fig. 4 shows that the filter is stable and able to work with rPPG signals from different algorithms. As a conclusion of this experiment, we could say that in an *intra-dataset* scenario, both sequence-to-sequence approaches outperform the results provided by the filters found in the literature; however, MTM turns out to be better than MTO, especially when it comes to improving the quality of the signal.

**Fig. 9:**
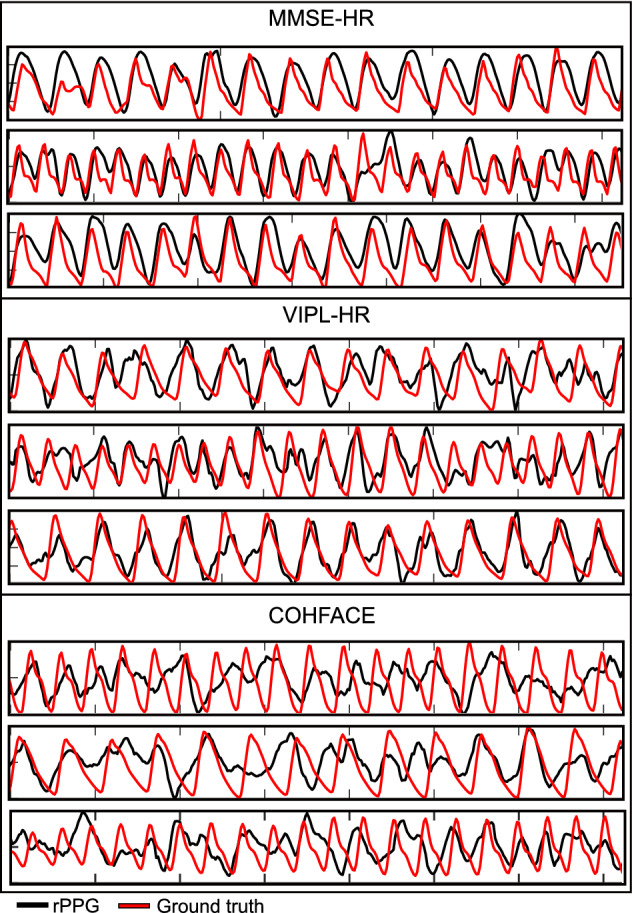
rPPG examples. Examples of rPPG signals from the three databases used: MMSE-HR, VIPL-HR, and COHFACE

The *cross-dataset* protocol is particularly interesting because based on Fig. 5, there is a dependence between the signals used during training and testing. This behavior may be because each dataset has rPPG signals of a specific quality. For example, the MMSE database has an rPPG-SNR average of $7.65\pm 4$ dB, indicating that the signals are mostly of good quality, VIPL-HR has a larger amount of data, and its rPPG-SNR average is $1.04\pm 4$ dB, COHFACE on the other hand, has an rPPG-SNR average of $-0.96\pm 4$ dB. This signal quality difference between the three datasets is also visible in Fig. 9. Therefore, if there is a dependence between the quality of the signals used for training and testing: first, training the network on high-quality signals (MMSE-HR) and testing on low-quality signals (COHFACE) or vice versa should give poor performance, and second, training on a broad spectrum of good and poor quality signals (VIPL-HR) should give good performance.

Analyzing the metrics in Fig. 5, we notice that when training in VIPL-HR and testing in MMSE-HR, LSTM-MTO performs better in *r*, SNR, and TMC than other filters, but in MAE, it is the second-best value after WV. LSTM-MTM improves the signal quality metrics but fails to outperform the other methods in the heart rate measurement. Training in VIPL-HR and testing in COHFACE shows how the LSTM-based filter outperforms the other filters. The MTM approach is the best of the two proposed. Training in MMSE-HR and testing in VIPL-HR allows the LSTM-MTM filter to present the best result in MAE, a value very close to the best values given by BP and WV in *r*, and the best results in SNR and TMC. LSTM-MTO, on the other hand, gives values close to the best values in MAE and *r* but decreases the values of SNR and TMC. Continuing with the training in MMSE-HR but testing in COHFACE, we can see that LSTM-MTO has the best results for the MAE and *r* metrics, but some values are lower in SNR and TMC. LSTM-MTM, on the other hand, has a contrary behavior, it improves the other filters in SNR and TMC, and although it improves the result of *r*, it does not manage to overcome the BP and WV filters in MAE. Therefore, neither of the two proposed methods is sufficiently robust to over-perform the four metrics.

LSTM-DF-MTM shows to be more sensitive to the noise of the training signals, this is visible when we test in VIPL and MMSE having trained in COHFACE, LSTM-DF-MTO on the other hand, is more stable in the same conditions.

Therefore, the *cross-dataset* scenario shows that for the LSTM-based filter, there is a dependence on training and test data related to the quality of rPPG signals, reflected in the rPPG-SNR value of the databases. We corroborated this hypothesis in the *rPPG-SNR* experiment.

Regarding the experiment *amount of training data*, in Fig 6, the many-to-one approach shows that when decreasing the number of data from 100% to 50% and 25%, MAE and *r* remain stable, when decreasing from 10% and 5%, MAE is stable but the value of *r* starts to decrease, finally when having 1% of data MAE and *r* decrease. Concerning signal quality metrics, the behavior is similar; MTO still has higher values than the classic filters in all configurations, although in the 5% and 1% of data, SNR and TMC show lower values than those present between 100% and 10% of data.

In HR measurement, the MTM filter shows a higher sensitivity to the amount of training data than the MTO. Even though the MAE value remains relatively constant, *r* starts to decrease from taking percentages equal to and less than 25%. In the case of signal quality metrics, the many-to-many approach shows a higher performance than many-to-one regardless of the amount of training data, and it is always higher than the conventional filters.

Both sequence-to-sequence approaches can be trained with a set similar to the VIPL005 (45 min, 90 signals) and start to obtain better results than conventional filters in a test set with similar rPPG-SNR values.

In the HR measurement metrics presented in the experiment *rPPG-SNR dependence* (Fig. 7), the MTO filter does present dependence on the rPPG-SNR during training; MAE values improve from VIPLAQ0 to VIPLAQ3, and the results decrease only a little in VIPLAQ4, *r* presents a similar behavior by having better results in the training sets with low values of rPPG-SNR than in the sets with the higher ones. In terms of signal quality, the variation in both SNR and TMC results is small, but the lower values agree with the cleaner VIPLAQ0 and the noisier VIPLAQ4.

In the MTM filter, the dependence is more evident. Related to HR measurement, MTM is more sensitive to train in signals with the lowest and highest indices of rPPG-SNR (VIPLAQ4 and VIPLAQ0, respectively) than the MTO approach. The highest performances are presented in VIPLAQ2 and VIPLAQ3 for MAE and *r*. In signal quality, SNR and TMC only seem to be sensitive to the training set VIPLAQ4 because for the other four training sets, their values are constant and even higher than those presented by the MTO approach. As a conclusion of the first part of this experiment, we can say that using the VIPLB test set whose rPPG-SNR average is $1.09\pm 4.28$ dB, the two best training sets are VIPLAQ2 and VIPLAQ3, which coincide as they are the closest in quality of the rPPG signals with an rPPG-SNR average of $1.89\pm 6.23$ dB and $0.73\pm 5.56$ dB, respectively. Therefore, there is a dependence with the rPPG-SNR average in the training and testing sets, specifically, the rPPG-SNR in the training set must be similar to that of the test set to have good results.

The second part of the experiment, *rPPG-SNR dependence* depicts in Fig. 8 that in MTO and MTM, results are better in the test set VIPLAQ0, and they degrade as the test set VIPLAQ4 is reached. The only exception is presented in TMC given by MTM, where the values are independent of the testing set. For the rest of the metrics, all values with their standard deviation get worse when the testing set gets noisier. In HR measurement, the performance of MTO decreases more abruptly than with MTM. On the other hand, interestingly, if the LSTM training can be ensured in a data set with a high and low average rPPG-SNR signal balance, there will be an improvement in the heart rate measurement and in the quality of the rPPG signals compared to classical filters.

## Conclusions

In this article, we analyzed the performance of an LSTM network for rPPG filtering using multiple protocols. Two sequence-to-sequence filter approaches were evaluated: many-to-one and many-to-many. We used three public databases in different experiments from which we can draw the following conclusions: the experiment *amount of training data* showed that a relatively low number of signals is needed to train the LSTM network efficiently. It was shown that even a dataset of approximately 90 signals totaling 45 min in total could be sufficient as a training set. In an intra-dataset scenario, both MTO and MTM over-perform the conventional filters, but it is the MTM approach that gives the best results.

We presented a comparison between the six state-of-the-art methods for rPPG estimation: PVM, POS, PbV, G-R, Chrom, and Green after using the LSTM-based filter. The results suggest stable values better than conventional filters, where using the LSTM-based filter on rPPG signals acquired by the PbV method gave the best performance.

The *cross-dataset* and *rPPG-SNR dependence* protocols are perhaps the most interesting experiments as they reflect a clear dependence of the LSTM-based filter on the rPPG-SNR average of the training and testing sets. Concerning this aspect, experiments showed that it is recommended that the rPPG-SNR average of the training set has to be as close as possible to those of the test set. This ensures the LSTM-based filter overperforms classical filters even in a cross-dataset scenario.

The experiments let us appreciate how an LSTM-based filter is a better alternative than the classical filters, which improves not only the heart rate measurement but also the quality of the signal. Finally, as future work, we are using the LSTM-based filter for rPPG signals acquired in the near-infrared NIR spectrum to improve the quality of the signals, and we will evaluate the benefits of our LSTM-DF to improve the performance of methods based on a precise analysis of rPPG signals, such as, for example, the analysis of HRV for the estimation of emotional states.

## Methods

### Data

We used the following three public databases in our experiments.

#### MMSE-HR

The Multimodal Spontaneous Emotion Corpus—Heart Rate database (MMSE-HR) [49] includes videos with many facial movements and expressions. The database has been built for further investigation in emotion recognition. Forty subjects are recorded performing 102 tasks, the resolution of each video is 1040 x 1392 pixels with a frame rate of 25 fps, the BIOPAC 150 data acquisition system was used to obtain the blood pressure ground truth at 1 kHz. The duration of each video is between 30 and 60 s.

#### VIPL-HR

The research in this paper uses the VIPL-HR database collected by the Institute of Computing Technology Chinese Academy of Sciences [22, 50]. The database contains 107 subjects recorded by three different instruments in nine scenarios: stable, motion, talking, dark, bright, long distance, exercise, phone stable, and phone motion. Although this database contains 752 near-infrared videos, we only consider the 2378 visible light videos, the resolutions of the videos are between 960 x 720 and 1920 x 1080, at 25 and 30 fps, respectively. The ground truth photoplethysmography signals were recorded using the CONTEC CMS60C BVP sensor at 60 Hz.

#### COHFACE

In the COHFACE database [51], four 1-min videos with different luminance were acquired from 40 subjects, generating 160 videos with a resolution of 640x480 pixels and a frame rate of 20 Hz. The BVP ground truth signals of photoplethysmography were acquired at 256 Hz.

#### Ground truth selection

In the databases used, due to the movement of the subjects, or failures in the acquisition devices, some ground truth signals present anomalies. These inconsistencies (gaps and false peaks) usually happen at the beginning and end of the acquisitions and less frequently during the measurement. However, due to the nature of the LSTM-based networks, it is not suggested to perform the training procedure with ground truth signals that present these types of problems. Therefore, a ground truth selection step is necessary. For this purpose, ground truth signals are checked individually to take only the continuous segment with a reliable ground truth morphology. In most signals, it was sufficient to remove the first and last seconds, while a considerable part of the signal was removed in a small group. In order to maintain the reproducibility of the studies carried out in this article, attached to this document are the supplementary files with the information of the signals that were cropped in each database (Additional files 1, 2, 3). Once only the reliable ground truth signals are selected; Table 7 contains the parameters related to rPPG signals acquired by the PVM method (PVM-rPPG) [13] from the three databases: MMSE-HR, VIPL-HR, and COHFACE. rPPG-SNR is the average of the SNR coefficient of rPPG signals. Figure 9 depicts examples of rPPG signals from the three databases used. Note how the quality of the acquired rPPG signals varies for each database. This is due to the nature of the scenarios where the videos were acquired for each database, i.e., luminance, movement, and other factors present during the acquisition.

**Table 7:** Parameters of the PVM-rPPG signals presented in the MMSE-HR, VIPL-HR, and COHFACE datasets

	MMSE-HR	VIPL-HR	COHFACE
rPPG-SNR [dB]	$7.65\pm 3.78$	$1.07\pm 4.25$	$-0.96\pm 4.17$
Duration [min]	64	1,114.5	166
Number of signals	98	2256	164

It is essential to mention that the VIPL-HR database was designed to estimate rPPG signals and contains a large amount of data during various scenarios. Due to this, its average rPPG-SNR is 1.07 dB. MMSE-HR and COHFACE, on the other hand, have a considerably smaller amount of data and differ from each other, being MMSE-HR the database with the best quality in its rPPG signals with an average rPPG-SNR of 7.65 dB, and COHFACE with an average rPPG-SNR of -0.96 dB, being the most challenging database, these negative values are perhaps due to the level of compression of the videos or the luminance in the scenarios.

### Protocols

In the following, we will describe the experiments carried out to validate the use of an LSTM network for filtering rPPG signals. We conducted a classical intra-database evaluation followed by a cross-dataset evaluation. Additionally, we present experiments to study in more detail the advantages and limitations of using an LSTM-based network for rPPG filtering. First, we demonstrate that it is not necessary to use a large amount of data to train the network successfully and that there is a clear dependence of the rPPG-SNR average in the rPPG signals during training. It is important to mention that there is no overlap between training and testing data in any of the experiments presented.

**Fig. 10:**
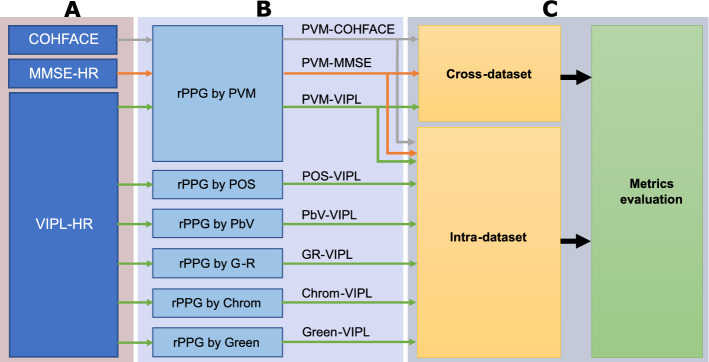
Intra- and cross-dataset rPPG evaluation. **A** Are the list of the databases used. **B** Are the rPPG measurement methods used, and **C** are the evaluation criteria along with the measurement of metrics

**Fig. 11:**
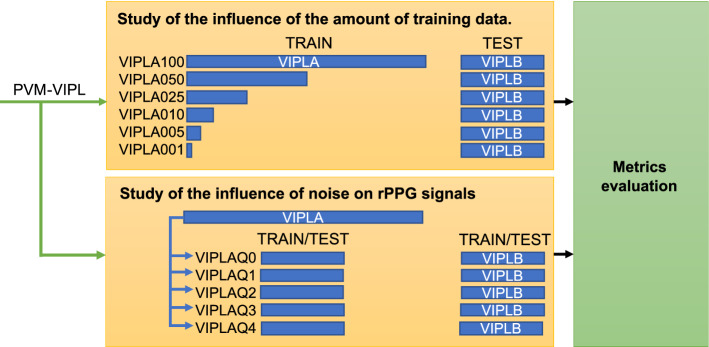
Protocols. PVM-VIPL is used to perform the protocols *amount of training data* (top panel), and *rPPG-SNR dependence* (bottom panel)

Figure 10 shows the distribution made in the databases used to study intra- and cross-dataset evaluations. A lists the databases used: COHFACE, MMSE-HR, and VIPL-HR. B contains the rPPG signals acquired by: PVM [13], POS [17], PbV [16], G-R [17], Chrom [18], and Green [3]. **C** presents the intra-dataset and cross-dataset evaluations with metrics measurement. Figure 11 resumes the PVM-VIPL signal generated in Figure 10, to be used in the study of the influence of the amount of data and noise on the rPPG signals during the training of the LSTM network, the protocol related to the amount of training is later called *amount of training data* (top panel), and the protocol related to noise is called *rPPG-SNR dependence* (bottom panel).

#### Intra-dataset

**Fig. 12:**
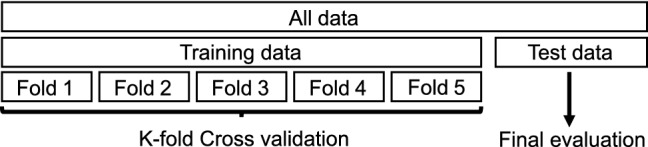
Evaluation. Fivefold cross-validation and final evaluation used in the *intra-dataset* protocol

In this standard evaluation protocol, we divided each dataset into 80% training and 20% testing to perform a final evaluation, within the 80% we developed k-fold cross-validation (CV for short) of fivefold, as shown in Fig. 12. In the first part of this experiment, we evaluated the performance of the LSTM-MTO and LSTM-MTM for the PVM-MMSE, PVM-VIPL, and PVM-COHFACE sets. In the second part, to analyze the filter robustness with rPPG signals acquired by different state-of-the-art methods, we chose the PVM-VIPL, POS-VIPL, PbV-VIPL, GR-VIPL, Chrom-VIPL, and Green-VIPL sets.

#### Cross-dataset

A realistic evaluation of the filter is to train the system using one database and to test it using other databases: this is the cross-dataset protocol. For this reason, the rPPG signals of the three sets, PVM-MMSE, PVM-VIPL, and PVM-COHFACE, were used individually as a training set to perform the test on the two remaining sets of data.

#### Amount of training data

In this experiment, we wanted to study the sensitivity of the LSTM-based filter to the amount of information given during training. A stratified train–test–split in PVM-VIPL was made, having the VIPLA and VIPLB sets with 80% and 20% of signals, respectively. Consequently, we used the VIPLB set as a testing set and VIPLA as the training one. This way, we take the set VIPLA and perform a stratified division at 100%, 50%, 25%, 10%, 5% and 1% of the data, calling these training sub-sets VIPLAx with x=[100,050,025,010,005,001]. Note that the set VIPLA100 is indeed the same VIPLA, but this notation is used only to appreciate the sub-sets characteristics easily. The stratified division guarantee sub-sets with a balanced rPPG-SNR. This last detail is important as we will see in *rPPG-SNR dependence*, that having training and testing sets with unbalanced average rPPG-SNR may change the performance of the network.

#### rPPG-SNR dependence

In this experiment, we wanted to simulate a scenario where the training and testing sets of the LSTM-based filter present a different signal quality given by the rPPG-SNR values. This scenario may happen when rPPG signals are estimated from different approaches, as we did in *cross-dataset*, and in the *intra-dataset* experiment with PVM-VIPL, POS-VIPL, PbV-VIPL, GR-VIPL, Chrom-VIPL, and Green-VIPL. In order to assign a label representing the level of the rPPG-SNR, the SNR average of each subject ($\text {SNR}_i$ with $i \in [1,N]$) was measured and assigned a label $e_i$: 0, 1, 2, or 3, where 0 indicates a low value of rPPG-SNR and 3 a high one. We relate the value of rPPG-SNR to the quality of the signals, and to define the rPPG-SNR thresholds for the labels, the maximum and minimum SNR values were taken from all subjects, and this range was divided among *h*-length 4 sub-ranges. This rPPG-label-assignment procedure is defined in equation 1:

1 $$\begin{aligned} e_i = {\left\{ \begin{array}{ll} 0 &{} \text {SNR}_i<\min (\text {SNR})+h, \\ 1 &{} \min (\text {SNR})+h \le \text {SNR}_i<\min (\text {SNR})+2 h, \\ 2 &{} \min (\text {SNR})+2 h \le \text {SNR}_i<\max (\text {SNR})-h, \\ 3 &{} \text {SNR}_i \ge \max (\text {SNR})-h, \end{array}\right. } \end{aligned}$$

where *h* is given by equation 2:

2 $$\begin{aligned} h = \frac{\text {max}(\text {SNR})-\text {min}(\text {SNR})}{4}. \end{aligned}$$

Using these labels, we generated five new sets VIPLAQi with i=[0,1,2,3,4]. Where VIPLAQ0 represents a set of data with signals mostly with good quality (high rPPG-SNR), the four remaining new sets decrease in quality until they reach VIPLAQ4, whose signals mostly have bad quality (low rPPG-SNR). The first part of this experiment consists of taking the VIPLAQi as training sets and VIPLB as a testing set, and in the second part, the VIPLB set was chosen as the training set to be tested in VIPLAQi.

### Model

**Fig. 13:**
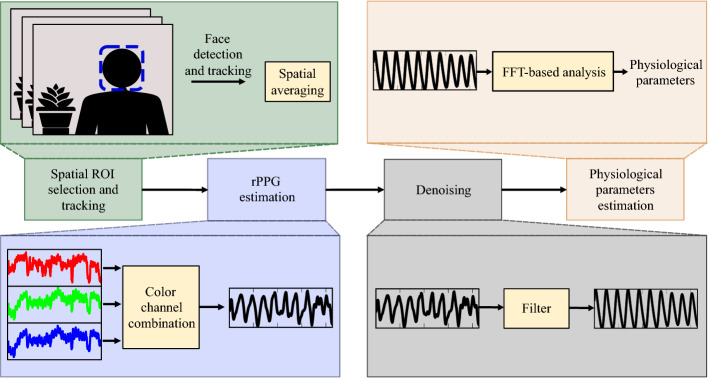
Framework. A spatial ROI selection and tracking video is performed, then the rPPG signal is estimated, and filtered by the MTO or MTM approach. Finally, the physiological parameters estimation is performed through a FFT-based analysis

To perform the rPPG signal filtering, we use the long-short-term deep-filter proposed in [47]. The workflow of the procedure is presented in Fig. 13. As a first step, we perform face tracking allowing us to take into account the pixels of the skin of the face only (ROI selection) followed by a spatial averaging process. Then, we use the RGB channels, and according to an rPPG estimation method, a color channel combination is performed. Afterward, the denoising of the rPPG signal is performed, and finally, the physiological parameter estimation on the filtered rPPG signal is computed through a fast Fourier-transform (FFT)-based analysis.

In the rest of this section, we will explain the LSTM-DF filter with its two approaches, MTO and MTM, followed by the procedure for organizing the databases so that they can be used to train the network.

#### Long short-term deep-filter

We need to extract the raw rPPG signal from the videos in the databases. Therefore, having one video with *T* frames, we compute a spatial ROI selection and tracking on the face of the subject to perform a spatial averaging between the pixels related to the skin. The result is a triplet of RGB values for each frame $r_{j}$, $g_{j}$, and $b_{j}$, where $j \in [1,T]$. Consequently, the vectors ${\mathbf {r}}$, ${\mathbf {g}}$, and ${\mathbf {b}}$ represent the RGB values for the full video. This way, being $\omega$ a color channel combination function applied by an rPPG estimation method, the rPPG vector ${\mathbf {y}}$ is presented in Eq. 3:

3 $$\begin{aligned} {\mathbf {y}} = \omega ({\mathbf {r}},{\mathbf {g}},{\mathbf {b}}). \end{aligned}$$

With the signal to be filtered, we use an LSTM network constituted by an input, a memory block, and an output. The memory block has the following parts: one *input gate* that learns which information is stored in the memory block, a *forget gate* that learns how much information is forgotten or retained from the memory block, and finally, an *output gate* that takes care of learning when the collected information can be used. We consider the rPPG filtering a sequence-to-sequence regression problem in univariate time series data. There are two LSTM network approaches explained below:

*Many-to-one (MTO)*: In this approach, multiple inputs allow to predict one single output, specifically, to forecast the next value based on the inputs. For this reason, we use a *L*-length sliding window with a step of one frame on the rPPG vector ${\mathbf {y}}$. Then, we use the LSTM-MTO filter $\varphi _o$ to obtain the filtered rPPG vector ${\hat{{\mathbf {y}}}}$ as shown in Eq. 4:

4 $$\begin{aligned} {\hat{y}}_{[j]} = {\left\{ \begin{array}{lc} y_{[j]} &{} j \in [1,L], \\ \varphi _o({\mathbf {y}}_{[j-L:j-1]};\theta ) &{} \text {others}, \end{array}\right. } \end{aligned}$$

where $\theta$ is the set of all gates parameters and weights already trained, and $j \in [1,T]$. Note how the first estimated sample given by the filter is actually at $L+1$.

*Many-to-many (MTM)*: The MTM approach uses multiple inputs to give multiple outputs, specifically the number of inputs is the same number of outputs. Similarly, as in MTO, we use a *L*-length sliding window with a step of one frame, to use the MTM filter as $\tilde{{\mathbf {y}}}(j) = \varphi _m({\mathbf {y}}_{[j:j+L-1]};\theta )$ with $j \in [1,T-L+1]$. The *L*-length outputs $\tilde{{\mathbf {y}}}(j)$ are later combined to create a single signal ${\hat{{\mathbf {y}}}}$ using the overlap-add procedure as presented in Eq. 5:

5 $$\begin{aligned} {\hat{y}}_{[j]} = \sum _{l=1,j-l+1>0}^{L}\tilde{{\mathbf {y}}}(j-l+1)_{[l]}. \end{aligned}$$

**Fig. 14:**
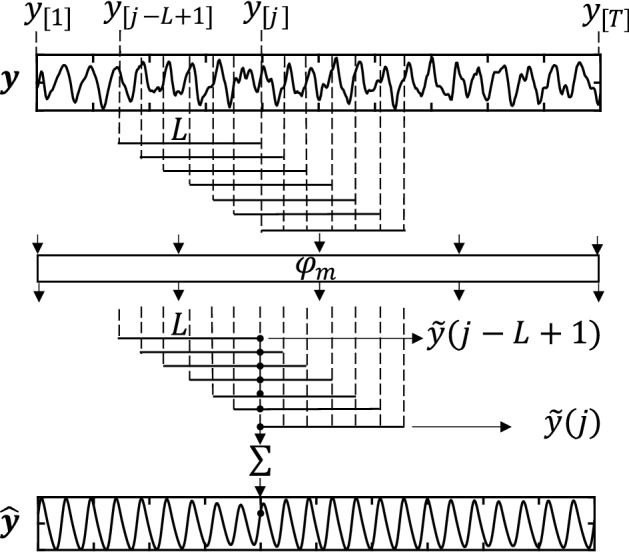
Overlap-add. Illustration of the overlap-add procedure computed after filtering rPPG signals by means of MTM ($\varphi _m$)

The process is illustrated in Fig. 14.

To train the MTO and MTM networks, we need more than one rPPG signal. Below we explain the process to create the training set from signals of different duration.

### rPPG training dataset building

To create the training set for the LSTM network, we consider the rPPG signal ${\mathbf {y}}_{i}$ related to the video *i* with length $T_{i}$, where $i \in [1,N]$ and *N* is the number of videos. Then, for the *N* videos, we use an *L*-length sliding window with a step of one frame to create the training matrices ${\mathbf {Y}}$ and ${\mathbf {G}}$ (vector ${\mathbf {G}}$ for MTO). In MTM and MTO, ${\mathbf {Y}}$ is composed of rPPG signals with a fixed length *L*. ${\mathbf {G}}$ represents the ground truth. In MTM, ${\mathbf {G}}$ is a matrix of PPG signals of same size than ${\mathbf {Y}}$. ${\mathbf {G}}$ is a vector in MTO approach. ${\mathbf {Y}}$ and ${\mathbf {G}}$ are given as the training set for the LSTM-based filter. The whole procedure mentioned is presented in Algorithms 1 and 2.
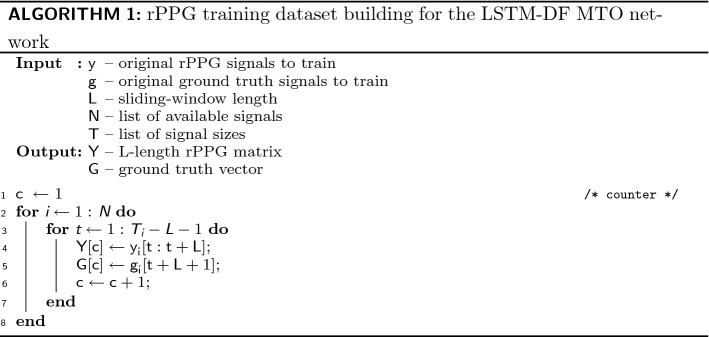

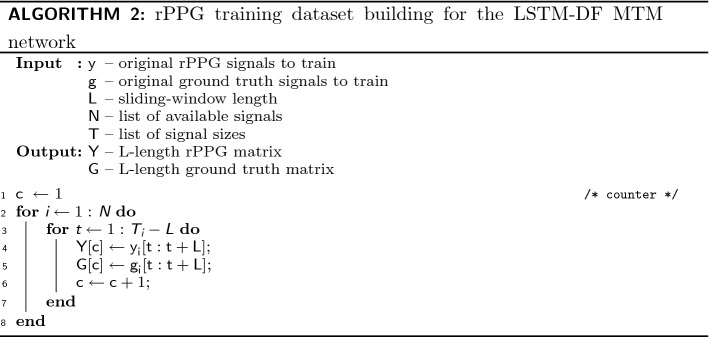


**Fig. 15:**
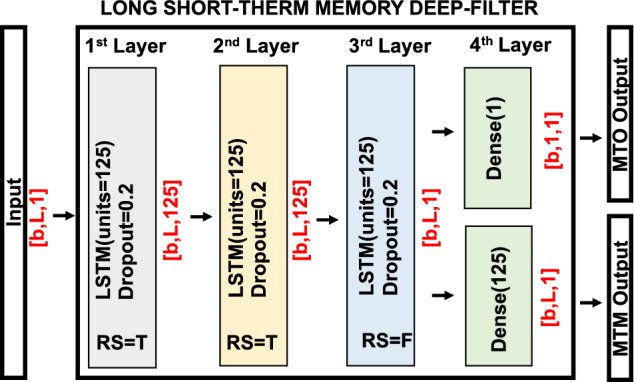
Network architecture. LSTM network architecture for MTO and MTM. Numbers in red represent the size of each data. RS=return sequences, T=true, F=false, b=batch size, L=sliding window length

With the input data and the neural network defined, all that remains is to know the architecture of the two approaches, MTO and MTM. Both architectures are shown in Fig. 15 where *L* is the input length, *b* is the batch size, and Return Sequence (RS) is an argument that decides whether a layer outputs each time step or its final time step. A dropout step is done on the first three layers to avoid the overfitting problem. The three LSTM layers contain 125 units.

### Evaluation metrics

#### Mean absolute error

The mean absolute error was calculated as the window-wise mean of the heart rate calculated using the contact-based ground truth waveform obtained by pulse oximeter (${\mathbf {Hc}}$), and the heart rate calculated using the rPPG signal (${\mathbf {Hr}}$). The MAE of the two vectors ${\mathbf {Hr}}$ and ${\mathbf {Hc}}$ of size *n* is presented in Eq. 6:

6 $$\begin{aligned} {\mathrm {MAE}}=\frac{1}{n} \sum _{j=1}^{n}\left| {\mathrm {{Hr}}_{j}}-{\mathrm {{Hc}}_{j}}\right| , \end{aligned}$$

where ${\mathrm {{Hr}}_{j}}$ and ${\mathrm {{Hc}}_{j}}$ are the value of ${\mathbf {Hr}}$ and ${\mathbf {Hc}}$ at position *j*, respectively.

#### Pearson correlation coefficient

Pearson correlation coefficient measures the linear correlation between vectors ${\mathbf {Hc}}$ and ${\mathbf {Hr}}$. A value of $r=1$ means a positive total linear correlation, while a $r=-1$ implies a negative linear correlation, finally, a $r=0$ indicates that there is no linear correlation between the estimations and the reference values. *r* is given by Eq. 7:

7 $$\begin{aligned} r=\frac{\sum _{j=1}^{n}\left( {\mathrm {{Hr}}_{j}}-{\overline{Hr}}\right) \left( {\mathrm {{Hc}}_{j}}-{\overline{Hc}}\right) }{\sqrt{\sum _{j=1}^{n}\left( {\mathrm {{Hr}}_{j}}-\overline{{Hr}}\right) ^{2}} \sqrt{\sum _{j=1}^{n}\left( {\mathrm {{Hc}}_{j}}-{\overline{Hc}}\right) ^{2}}}, \end{aligned}$$

where ${\overline{Hr}}$ and ${\overline{Hc}}$ are the averages of ${\mathbf {Hr}}$ and ${\mathbf {Hc}}$, respectively.

#### Signal-to-noise ratio

The SNR is a measure used to compare the power of a signal ${\bar{P}}_{{\mathrm {signal}}}$ with the power of the background noise ${{\bar{P}}_{{\mathrm {noise}}}}$. This metric is used to characterize the signal quality and it is calculated in Eq. 8:

8 $$\begin{aligned} {\mathrm {SNR}}=\frac{{\bar{P}}_{{\mathrm {signal}}}}{{\bar{P}}_{{\mathrm {noise}}}}, \end{aligned}$$

where the power average is given by ${\bar{P}}$ [52], results are given in decibels (dB). The SNR metric is performed regarding full-length signal.

#### Template match correlation

The template match correlation [53] is also used as an rPPG signal quality assessment metric. The following steps are performed regarding full-length signals:

1. The signal peaks are detected.
2. The median beat-to-beat interval is calculated.
3. With a window width equal to the median beat-to-beat interval, all pulses are extracted individually centered on their respective peak.
4. The template is calculated as the average of all the pulses.
5. The TMC coefficient is calculated as the average of the correlation of all the pulses with the template.

A value close to ${\mathrm {TMC}}=1$ means that the pulse shape of the evaluated or filtered signal is uniform, and therefore close to the expected signal, while a value close to ${\mathrm {TMC}}=0$ indicates the contrary.

### Implementation details

For the face detection procedure, we used the deep-learning-based OpenCV model, used in the Single Shot MultiBox Detector method implemented by Liu *et al.* [54]. Then, the Conaire *et al.* algorithm was used to select the skin pixels [55]. All ground truth and rPPG signals were re-sampled at the same frequency (25 Hz), then a detrending procedure was made followed by a normalization between -1 and 1. Finally, all ground truth signals were smoothed by the *smooth* *SciPy* function^1^. Classic filters were set as follows: bandpass filter with cutoff frequencies at 0.7 and 3.5 Hz, wavelet-based filter using the same parameters as in [31], and the Savitzky–Golay filter with a 9-samples window length and a polynomial order of two^2^ .

We used a personal computer with the following features: Intel Xeon 2.4 GHz CPU, 16 GB RAM, and a Graphics Processing Unit (GPU) NVIDIA GeForce RTX 2070 GPU. The LSTM network was implemented with the following libraries: Keras (v.2.2.4) with Tensorflow (v.2.0) backend. The activation functions for the LSTM and Dense layers were hyperbolic tangent and linear, respectively. The loss function was mean squared error, the first two LSTM layers had a dropout equal to 0.2, and the Adam optimizer was set with a learning rate scheduler of 0.002 between 1 and 10 iterations, 0.001 between 11 and 20, 0.0005 between 21 and 50, and 0.0001 for 51 or more iterations. The Glorot uniform initializer, also called Xavier uniform initializer, was used for the random weights initialization. The number of epochs used was 100, with a batch size of 32, following the same procedure explained in [47]. The sliding window used during the training setup had length $L=125$ frames, equivalent to 5 s. The network training during the final evaluation took for each dataset approximately: 1 h for the MMSE-HR, 14 h for VIPL-HR, and 3 h for COHFACE. The process of filtering on a 1-min signal takes approximately 1 s.

## Supplementary Information

**Additional file 1.** Table with the names of the subjects in the MMSE-HR database and their reliable segments.

**Additional file 2.** Table with the names of the subjects in the VIPL-HR database and their reliable segments.

**Additional file 3.** Table with the names of the subjects in the COHFACE database and their reliable segments.

## Data Availability

The datasets used and/or analyzed during the current study are available from the corresponding author on reasonable request. All data analyzed during this study can be replicated based on the original datasets along with the files included in this published article.

## Abbreviations

ECG: Electrocardiography
PPG: Photoplethysmography
rPPG: Remote photoplethysmography
HR: Heart rate
HRV: Heart rate variability
PCA: Principal component analysis
ICA: Independent component analysis
EVM: Eulerian video magnification
PVM: Pulse rate variability measurement
PbV: Blood-volume pulse vector
POS: Plane-orthogonal-to-skin
G-R/GR: Green and red image channels
Chrom: Chrominance-based rPPG
ROI: Region of interest
BP: Bandpass filter
WV: Wavelet filter
SG: Savitzky–Golay filter
RNNs: Recurrent neural networks
LSTM: Long short-term memory
LSTM-DF: Long short-term memory deep-filter
LSTM-MTO/MTO: Long short-term memory deep-filter, many-to-one
LSTM-MTM/MTM: Long short-term memory deep-filter, many-to-many
MAE: Mean absolute error
r: Pearson coefficient
SNR: Signal-to-noise ratio
TMC: Template match correlation
Hc: Contact-based heart rate
Hr: Remote-based heart rate
MMSE-HR: Multimodal spontaneous emotion corpus—heart rate database
VIPL-HR: Visual Information Processing and Learning—heart rate database
Hz: Hertz
dB: Décibels
GPU: Graphics processing unit
CV: Cross-validation
rPPG-SNR: SNR level in rPPG
PVM-rPPG: rPPG signal acquired by PVM method
PVM-MMSE: rPPG signal acquired by PVM method in MMSE-HR
PVM-VIPL: rPPG signal acquired by PVM method in VIPL-HR
PVM-COHFACE: rPPG signal acquired by PVM method in COHFACE database
POS-VIPL: rPPG signal acquired by POS method in VIPL-HR
PbV-VIPL: rPPG signal acquired by PbV method in VIPL-HR
GR-VIPL: rPPG signal acquired by GR method in VIPL-HR
Chrom-VIPL: rPPG signal acquired by Chrom method in VIPL-HR
Green-VIPL: rPPG signal acquired by Green method in VIPL-HR

## Footnotes

1 https://scipy.github.io/old-wiki/pages/Cookbook.html..

2 https://bit.ly/37DSVPY.
